# Microwave-Assisted Drying for the Conservation of Honeybee Pollen

**DOI:** 10.3390/ma9050363

**Published:** 2016-05-12

**Authors:** Angelo Canale, Giovanni Benelli, Antonella Castagna, Cristina Sgherri, Piera Poli, Andrea Serra, Marcello Mele, Annamaria Ranieri, Francesca Signorini, Matteo Bientinesi, Cristiano Nicolella

**Affiliations:** 1 Department of Agriculture, Food and Environment, Università di Pisa, via del Borghetto 80, Pisa 56124, Italy; angelo.canale@unipi.it (A.C.); g.benelli@sssup.it (G.B.); antonella.castagna@unipi.it (A.C.); cristina.sgherri@unipi.it (C.S.); ppoli@agr.unipi.it (P.P.); andrea.serra@unipi.it (A.S.); marcello.mele@unipi.it (M.M.); anna.maria.ranieri@unipi.it (A.R.); 2 Interdepartmental Research Center “Nutraceuticals and Food for Health”, Università di Pisa, via del Borghetto 80, Pisa 56124, Italy; 3 Consorzio Polo Tecnologico Magona, via Magona snc, Cecina (LI) 57023, Italy; f.signorini@polomagona.it; 4 Dipartimento di Ingegneria Civile e Industriale, Università di Pisa, Largo Lucio Lazzarino 2, Pisa 56122, Italy; c.nicolella@ing.unipi.it

**Keywords:** microwaves, honeybee pollen, *Apis mellifera*, flavonoids, freeze-drying, rutin, polyphenols, proline

## Abstract

Bee pollen is becoming an important product thanks to its nutritional properties, including a high content of bioactive compounds such as essential amino acids, antioxidants, and vitamins. Fresh bee pollen has a high water content (15%–30% wt %), thus it is a good substrate for microorganisms. Traditional conservation methods include drying in a hot air chamber and/or freezing. These techniques may significantly affect the pollen organoleptic properties and its content of bioactive compounds. Here, a new conservation method, microwave drying, is introduced and investigated. The method implies irradiating the fresh pollen with microwaves under vacuum, in order to reduce the water content without reaching temperatures capable of thermally deteriorating important bioactive compounds. The method was evaluated by taking into account the nutritional properties after the treatment. The analyzed parameters were phenols, flavonoids, with special reference to rutin content, and amino acids. Results showed that microwave drying offers important advantages for the conservation of bee pollen. Irrespective of microwave power and treatment time, phenol and flavonoid content did not vary over untreated fresh pollen. Similarly, rutin content was unaffected by the microwave drying, suggesting that the microwave-assisted drying could be a powerful technology to preserve bioprotective compounds in fresh pollen.

## 1. Introduction

Pollen is an important source of proteins and free amino acids for the diet of honeybees, *Apis mellifera* L. (Hymenoptera: Apidae). Honeybees use pollen as a protein supplement in the diet of the larvae of workers and drones, older than three days old, as well as to feed the adults of the same caste [1]. Besides the nitrogenous substances, present in the form of complex proteins or free amino acids, the pollen is also composed of water (about 16%), sugars (about 37%) and other substances (about 5%: lipids, vitamins of group B, provitamin a, folic acid, minerals) [2].

The commercial production of pollen is carried out by beekeepers, using special traps placed at the entrance of the hives. Honeybees are forced to pass through a grid with holes of a diameter specially calibrated, and their pollen load falls in a drawer underneath, which is emptied daily by the beekeeper (this type of pollen is internationally defined as “fresh pollen”). The threshold for the use of pollen traps is 10%–15% of the total pollen load harvested by bees; therefore, from a hive, about 4–5 kg of pollen are obtained within a year [3].

Pollen is gaining growing attention as a potential source of energy and proteins for human consumption. Currently, many countries (e.g., Brazil, Poland, Bulgaria, Switzerland) have established guidelines about the physical, chemical and microbiological standards that the pollen for human consumption must fit [2]. Besides this, each type of pollen has specific nutritional characteristics that reflect its botanical origin [4]. However, the conditioning carried out on the fresh pollen collected by bees before storage for human consumption can change its nutraceutical value, determining a quantitative alteration of the chemical composition. These changes are usually due to the processes of cleaning, dehydration, packaging and conservation applied to fresh pollen from beekeepers to increase the pollen shelf-life [5].

In particular, to reduce the water content of honeybee-collected pollen, the fresh pollen is subjected to conditioning processes (*i.e.*, artificial drying) with a few standardized methods [6,7,8].

From a technological point of view, the knowledge of the different factors contributing to the production of high quality dried pollen is scarce. Currently, the drying process is conducted at low temperatures, with short exposure times, in order to avoid the risk of Maillard compound formation. When the temperatures and the dehydration times are, respectively, too high or prolonged, the content of free amino acids strongly decreases (<2 g/100 g) with an appreciable reduction in the organoleptic value and the production of undesirable aromatic compounds, largely arising from the degradation of the S-methyl methionine [7]. Furthermore, a marked effect of the drying process on the reduction of the final content of antioxidants such as polyphenols and flavonoids has been highlighted, with consequent reduction of the nutraceutical value of the final product [8].

In this scenario, the optimization and standardization of the technologies employed to process the fresh pollen is crucial, since novel and reliable tools may help to increase the pollen shelf life and reduce the nutraceutical value alterations. Here we propose a microwave-assisted process as a novel tool to reduce the water content in Castanea sativa pollen for human consumption. The new method implies irradiating the fresh pollen with microwaves under vacuum, in order to reduce the water content without reaching temperatures capable of thermally deteriorating important bioactive compounds. The effectiveness of the method was assessed analyzing the nutritional properties of pollen, namely phenols, flavonoids and amino acids, after the drying treatment.

The obtained results are extremely encouraging, showing little or no effect of the treatment on the nutritional content of the pollen.

## 2. Results

The main results of the MW drying experimentation are reported in this section. All analytical results are compared with the untreated pollen sample (UP).

### 2.1. Final Temperature and Moisture Content

As described in the following Section 4.2, the temperature of the sample dried with microwaves under vacuum is measured immediately after the end of the treatment both near the top and near the bottom of the sample holder.

After the treatment, the weight loss of the sample is determined gravimetrically. Moreover, the moisture content is determined through thermogravimetric analysis (TGA).

Four main tests were carried out, varying the microwave power and the treatment time. The conditions and the results are reported in Table 1.

There is a significant difference between the top and bottom final temperatures. This is due to the fact that moisture is first removed by the bottom of the sample, where dry air is sent, and transported towards the top. At a certain point, a lower portion of the sample will contain very little residual moisture, and the temperature will then be allowed to rise above the water boiling temperature at the working pressure (33 °C at 50 mbar). At the end of the test, the temperature of the dried bottom is well over the water boiling temperature for all the tests; in contrast, at the top, residual moisture is still present in significant amounts, and the temperature is thus lower than or slightly above the water boiling temperature.

**Figure 1:**
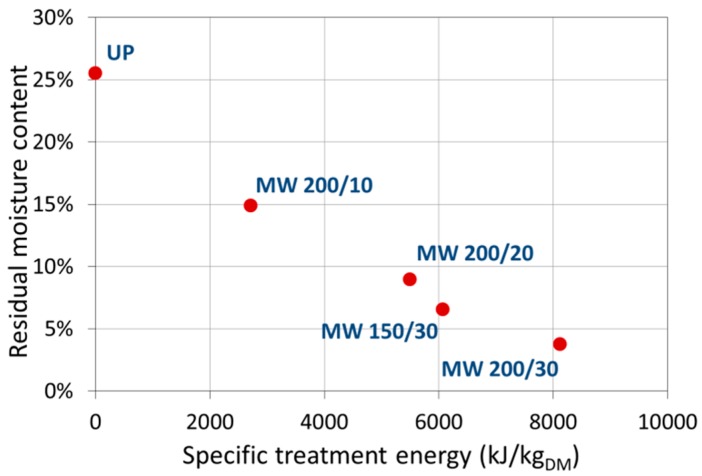
Decrease of residual water content in the pollen samples after the microwave drying treatment as a function of the total delivered MW energy.

As shown in Figure 1, as expected, by increasing the delivered MW energy (normalized with respect to the dry matter weight of the sample), a quasi-linear decrease in the pollen residual moisture content is obtained, while MW power seems to have little effect.

### 2.2. Biochemical Analysis

**Figure 2:**
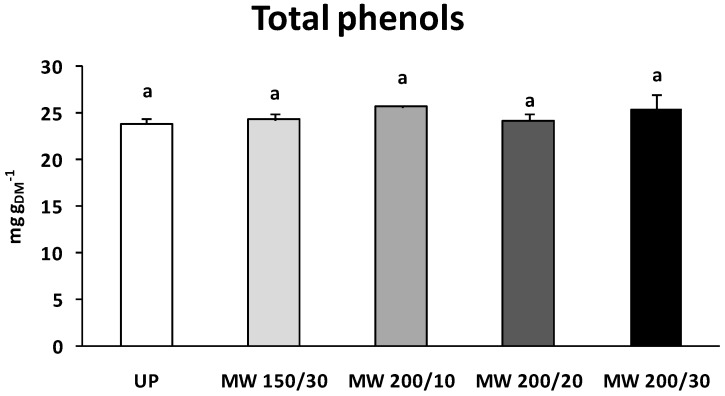
Total phenol content after the microwave drying tests; results are compared with that of the initial untreated pollen sample UP. Data represent the average of three independent measures ± SE (standard error). Different letters indicate significant differences according to one-way ANOVA followed by Tukey’s test (*p* ≤ 0.05).

The microwave drying treatment did not negatively affect total phenols content (*p* = 0.549) which showed instead slight, though not significant, increases in dried samples in comparison to untreated pollen, ranging from 1.5% (MW 200/20) to 7.9% (MW 200/10) (Figure 2).

**Figure 3:**
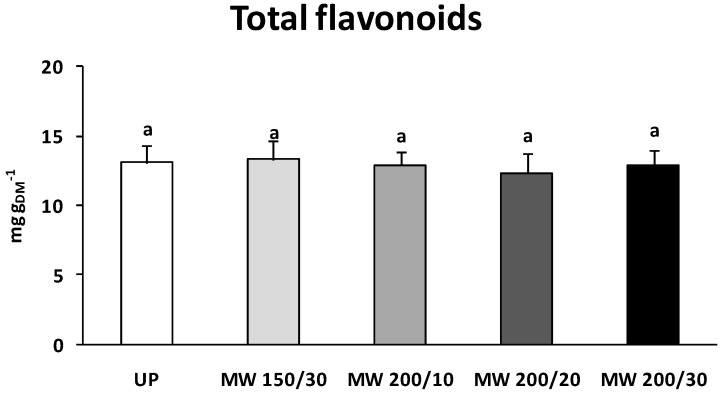
Total flavonoid content after the microwave drying tests; results are compared with that of the initial untreated pollen sample UP. Data represent the average of three independent measures ± SE. Different letters indicate significant differences according to one-way ANOVA followed by Tukey’s test (*p* ≤ 0.05).

Figure 3 reports the flavonoids content of the untreated pollen sample and of the microwave-dried pollen samples. Similarly to the observations in the case of total phenols, the content of total flavonoids, which accounted for about 50% of total phenols, was not significantly influenced by the microwave drying treatment (*p* = 0.981). Percentage of variation with respect to the untreated sample ranged from −6.0% to +1.7%.

The same extracts used for phenols and flavonoids analyses were used to quantify rutin content (Table 2). Again, rutin was unaffected by the microwave treatment, at any microwave power and treatment time (*p* = 0.465). Following drying, rutin content underwent a slight increase (2%–9%, depending on drying conditions), even if changes were not statistically significant.

As in the case of free proline, total proline remained unchanged in the MW 150/30 treatment in comparison with the fresh sample but a significant decrease was monitored after the MW 200 treatments (*p* = 0.0003). As a result of the changes in the free and total proline contents, their relative ratios showed a significant increase (*p* = 0.0036) in the MW 200/20 and MW 200/30 treatments, indicating an increase in free proline compared to proline contained in proteins. It is noteworthy that proline contained in the free amino acid fraction represented about the 40% of the total proline content. Free amino acids reached a value of about 27 mg/g_DM_ in the fresh sample and did not change after the MW 150/30 and MW 200/30 treatments. In contrast, they underwent a significant decrease (*p* = 0.000) following the MW 200/10 and MW 200/20 treatments. Total amino acid content approached the 30% of pollen composition in the fresh sample and was always subjected to a significant decrease after treatment (*p* < 0.0001). The relative ratio free to total amino acids, indicative of the release of amino acids from proteins, underwent to a significant increase with treatments (*p* = 0.0036). Free proline was the main component of the free amino acid fraction ranging from 64% to 72%. This percentage was subjected to a significant decrease (*p* = 0.02) only after the MW 200/20 treatment (Table 3).

## 3. Discussion

Honeybee pollen contains variable amounts of polyphenols, depending on the plant and the geographical origin as well as on the collecting season. Serra Bonvehí *et al.* [8] report a minimum content not lower than 1.20 g/100 g for most of the eleven Spanish samples analyzed. The same samples contained 0.35–0.78 g/100 g of total flavonoids. Higher phenols and flavonoids contents, ranging from 1.3 to 8.2 g/100 g, were measured in Polish honeybee pollen deriving from both herbaceous and tree species [9]. In accordance with these reports, in the present research, untreated fresh pollen contained about 2.4 g/100 g phenols and 1.3 g/100 g flavonoids. Flavonoids and other phenolic compounds [2,9], play a key preventive action against the onset of cardiovascular and neurodegenerative diseases. Cancer and age-related diseases [10,11] are the main determinants of antioxidant activity displayed by honeybee pollen. According to Serra Bonvehí *et al.* [8], 20 mg/100 g is the minimum quantity of rutin necessary to meet the nutritional and biological properties of bee pollen required in the European market. In this study, rutin content of untreated *C. sativa* pollen contained about 32 mg/100 g. Among free flavonoids, rutin is particularly sensitive to conditioning processes of fresh pollen, often carried out at elevated temperatures, as well as to long storage periods. The rutin content can therefore represent a marker of quality for honeybee pollen.

The nutraceutical quality of honeybee pollen, besides being dependent on the floral and geographical origin, is strictly influenced by process treatments, such as washing and drying. In the present study, irrespective of microwave power and treatment time, phenol and flavonoid content did not vary in respect to untreated fresh sample. Similarly, rutin content was unaffected by the microwave drying, suggesting that this process could be a powerful technology to efficiently preserve bioprotective compounds.

Besides containing healthy antioxidant compounds belonging to the flavonoid class, bee pollen is also important because it is a nutritional source of proteins as well as of free amino acids. Even if each pollen has specific characteristics mainly linked to the floral species, values found in *C. sativa* pollen relative to the contents in total free amino acids, free proline and the percentage of free proline on the total free amino acids were comparable to those detected by Serra Bonvehí *et al.* [8] in 11 different samples of bee pollen, demonstrating once again that proline was the main represented amino acid. Similarly, to the range reported by Campos *et al.* [2] for protein contents, our results showed values from 20% to 33% of total amino acid contents.

The shelf life of a pollen depends on water content and it is important to perform a correct drying to maintain nutritional as well as organoleptic qualities. Among the others, composition in free amino acid is affected by drying in honeybee pollen. A minimum quantity of 2% of free amino acid content is suggested to standardize the commercial pollen in the European market [8] and with all the microwave treatment tests we performed this value was maintained. In addition, the proline index value (free proline to free amino acid ratio) was always below 80%, indicating that microwave treatment did not affect the nutritional value of the bee pollen [6]. However, following microwave-assisted drying treatments, a decrease in total amino acid content and an increase in free to total amino acid ratios were observed in our experiments. However, given the small variations in the concentration of the nutritional parameters, it was not possible to individuate a trend between the energy delivered during the MW treatment and the variation of these parameters.

Overall, these results were indicative of the fact that microwave treatment likely induced protein degradation and/or proteolysis, anyway, without altering main commercial indexes for pollen quality.

Further tests will be conducted in order to evaluate the optimal operating conditions for the MW drying treatment. Moreover, the biological analysis will be carried out on the treated samples after 3 and 6 months, with the objective of evaluating the effect of the new treatment on the shelf life of the pollen.

## 4. Materials and Methods

### 4.1. Pollen Samples

Tests were conducted on honey-bee collected *C. sativa* pollen harvested by a beekeeper in July 2015 in chestnut grows located in Castelnuovo Garfagnana (44°06′22.7″N 10°24′02.7″E, Lucca, Italy), by using a pollen trap (Metalori, Italy). Samples were stored at −20 °C and transferred to the laboratories within an hour for further conditioning treatments. Morphological analyses were performed on samples, and the dominant pollen was unambiguously identified as *C. sativa* pollen. Post-conditioning, all analytical results were compared with the fresh untreated pollen sample (UP).

### 4.2. Microwave Drying

**Figure 4:**
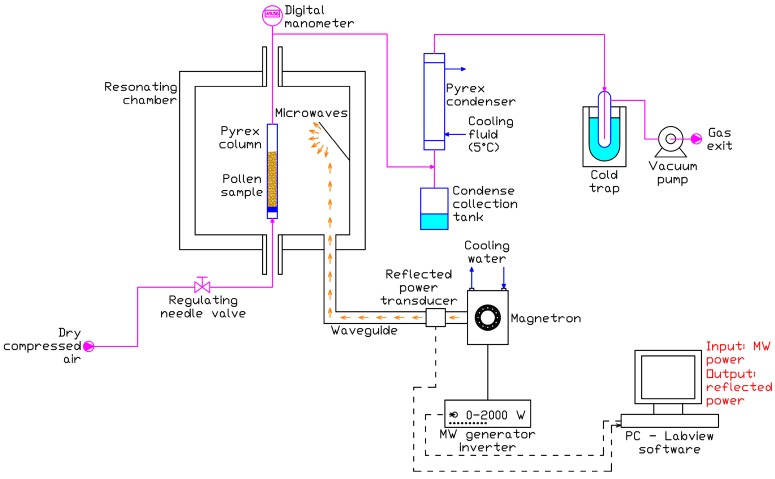
Schematic of the microwave drying experimental set-up.

The schematic of the microwave (MW) drying experimental set-up is shown in Figure 4.

The core of the set-up is a microwave oven with a 600 × 600 × 600 mm resonating chamber made of stainless steel. Microwaves, at a frequency of 2.45 MHz, are generated by a water cooled magnetron and fed to the chamber through a waveguide; the magnetron power is regulated in the range 0–2000 W by an inverter, and a transducer measures the MW power reflected towards the magnetron. Through a PC equipped with a Labview application, the user is allowed to select the MW power and to collect the reflected power data.

The pollen sample is contained into a Pyrex column (sample holder) which is introduced into the resonating chamber; dry air from a compressor is fluxed at 100 L_STP_/h in the Pyrex container bottom through a rotameter equipped with a regulating valve; the air is fed at room temperature (about 20 °C). The condensable gas generated during the treatment with MW (mainly steam) and transported by the air stream is condensed in a Pyrex condenser downstream the sample holder, and the condense is collected in a flask. The gas stream is then sent to a vacuum pump (after passing a liquid nitrogen cold trap) that maintains vacuum in the sample holder; the vacuum is measured by a digital manometer immediately downstream the sample holder.

**Figure 5:**
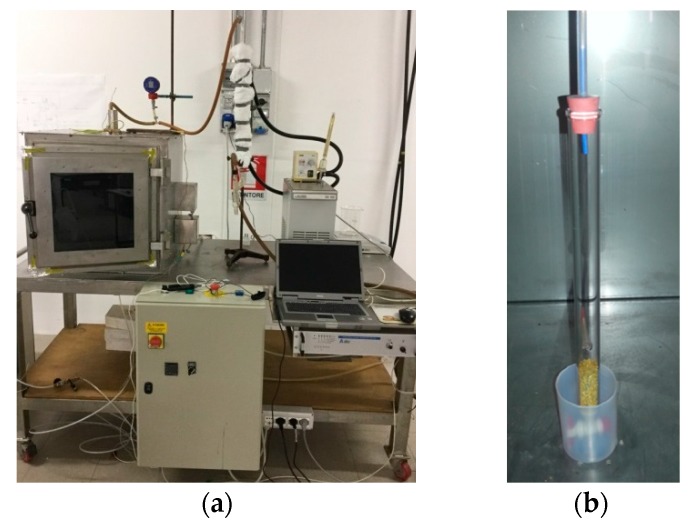
(**a**) Picture of the microwave drying experimental set-up; (**b**) Picture of the sample holder.

The experimental set-up is shown in Figure 5a. The sample holder is a Pyrex column with total capacity equal to 100 mL, diameter 25 mm and height 270 mm. In the column rubber cap, a hole is realized for gas exit, while dry air enters the bottom of the column via a valve (Figure 5b).

The tests were conducted at the absolute pressure of 50 mbar. This pressure was chosen due to the following considerations:

- MW absorption by the pollen sample and the following heating of pollen is due almost exclusively to the presence of water in the sample;
- at 50 mbar, the boiling point of water is around 33 °C, which is a sufficiently low temperature in order to avoid thermal degradation of the sample;
- we can assume that once the sample reaches the boiling point of water during MW irradiation, much of the electromagnetic energy is converted to latent heat for water vaporization, and that the temperature increase significantly slows down.

The main objective of the designed treatment is to decrease the water content of the sample without exposing it to excessively high temperatures. Both the choice of the working pressure and of moderate MW power were made on this basis. Moreover, the stream of dry air is added to facilitate the transport of vaporized water away from the sample.

Hereafter, the exact testing procedure is reported:

- a sample of about 50 g of pollen (stored at −20 °C in a freezer) is weighed and inserted into the sample holder;
- the sample holder is inserted in the MW oven chamber, and connected with the air tube and the evacuation tube;
- the vacuum pump is turned on and the air valve is regulated in order to reach a steady pressure of 50 mbar in the sample holder;
- the MW oven is turned on and the MW power maintained at the desired value for a given treatment time;
- at the end of the treatment, the MW is turned off, the air valve closed and the vacuum pump stopped;
- the pollen sample is immediately weighed and its temperature measured with a K-type thermocouple;
- the sample is then transferred into an airtight container and stored in freezer at −20 °C, waiting for the analyses.

### 4.3. Thermogravimetric Analysis

Each sample of both untreated and MW dried pollen is mixed thoroughly and three different smaller portions, each weighting some milligrams, are taken and used for the determination of water content, through thermogravimetric analysis (TGA).

The analysis was conducted using nitrogen at 100 mL/min as carrier gas, and with a temperature ramp from 20 to 120 °C, at a rate of 10 °C/min. The moisture content was taken as the average value of the three analysis.

The instrument used was a TGA Pyris 1 Perkin Elmer (Waltham, MA, USA).

### 4.4. Amino Acid and Proline Extractions

For the determination of free amino acids and free proline, pollen (0.1 g) was extracted with 80% ethanol by mortar and pestle. After sonication for 5 min, samples were centrifuged at 12,100 *g* for 15 min. The operation was repeated once again and the supernatants were vacuum dried. Samples were dissolved in MilliQ water and filtered by Sartorius (Goettingen, Germany) filters (Minisart 0.45 μm) to remove any suspended material.

For the determination of total amino acids and total proline, pollen (0.1 g) was incubated in 6 N HCl at 110 °C for 24 h. After filtration by Sartorius (Goettingen, Germany) filters (Minisart 0.45 μm), some aliquots of samples were vacuum dried and resuspended in MilliQ water.

### 4.5. Proline Determination

The proline content in the extracts from free and total amino acid determinations was detected following the method of Magné and Larher [12]. This procedure allows elimination of carbohydrate interference during proline assays and results suitable in case of food products that contain soluble carbohydrates. Briefly 2 mL of 1% ninhydrin in glacial acetic acid (60%, v/v) were added to samples (0.5 mL). After shaking, samples were taken in a boiling water bath for 1 h. Then 5 mL toluene were added in order to extract the chromophore. After 15 s of vigorous shaking the phases were allowed to separate and the absorbance of the upper phase was read at 520 nm. Calculations were performed by using a calibration curve with proline standard in the range 3–30 µg.

### 4.6. Amino Acid Determination

Amino acids from free and total amino acid extracts were detected following a method developed by Magné and Larher [12], but substantially modified. A 0.2 M citrate (Na^+^) buffer was prepared from analytical grade citric acid and was added with 8 mM SnCl_2_. A 4% ninhydrin solution was made with ethylene glycol. Samples (0.1 mL) were added of 1 mL of buffer and 1 mL of ninhydrin solution. After shaking, the test tubes were heated in a boiling water bath for 20 min. Then the tubes were cooled to room temperature and 5 mL of 50% (v/v) isopropanol were added as a diluent. Absorbance of the chromophore was read after 15 min at 570 nm. Calculations were performed by using a calibration curve with leucine standard in the range 3–30 µg. The relative color yield for proline of the ninhydrin reaction at 570 nm was reported as zero. For this reason, free and total amounts of amino acids were calculated by the sum of amino acids detected at 570 nm and proline from extracts for free and total amino acids determination, respectively.

### 4.7. Phenolic Compound Extraction

Samples (0.5 g) were extracted using a total of 15 mL of methanol:water (80:20 v/v). Solution was sonicated in ultrasonic bath for 30 min and then extracted for 30 min on a magnetic stirrer at 4 °C. The supernatant was recovered by centrifugation (15 min, 14,000 *g*) at 4 °C. The extraction procedure was repeated two additional times without the sonication step. The collected extracts were pooled together and filtered with 0.45 μm Minisart filters (Sartorius Stedim Biotech, Goettingen, Germany).

### 4.8. Quantification of Total Phenols and Flavonoids

Total phenols were determined using the Folin–Ciocalteu method, modified as described by Barbolan *et al.* [13]. The reaction mix was composed by 1.85 mL of distilled water, 0.125 mL of Folin-Ciocalteu reagent, 0.5 mL of a 20% Na_2_CO_3_ solution and 25 μL of extract. After 30 min of incubation at room temperature, the absorbance was recorded at 750 nm. Total phenols were expressed as milligram of gallic acid equivalents 100 g^−1^ DW.

Total flavonoids were determined as described by Kim *et al.* [14], by mixing 60 μL of 5% NaNO_2_, 40 μL of 10% AlCl_3_, 400 μL of 1M NaOH and 100 μL of extract. The solution was diluted with 200 μL of distilled water and the absorbance was recorded at 510 nm. The flavonoid concentration was expressed as milligram of catechin equivalents 100 g^−1^ DW.

### 4.9. Rutin Quantification by HPLC Analysis

The analysis was performed by a Spectra System P4000 HPLC equipped with a UV 6000 LP photodiode array detector (Thermo Fisher Scientific, Waltham, MA, USA) using a Phenomenex Prodigy LC-18 RP column (5 µm particle size, 250 × 4.6 mm, Phenomenex Italia, Castel Maggiore, Italy). Elution was carried out at a flow-rate of 1 mL·min^−1^ using 5% formic acid in water as solvent A, and 5% formic acid in methanol as solvent B, according to the following gradient: 0–5 min 95% A, 5–10 min 95%–88% A, 10–13 min 88% A, 13–35 min 88%–71% A, 35–50 min 71%–54% A, 50–52 min 54%–20% A, 52–57 min 20% A, 57–60 min 20%–0% A, 60–70 min 0%–95% A, followed by 5 min re-equilibration in the initial condition before the next injection. Detector was set at 340 nm. Rutin was quantified using standard curve of commercial standard (Sigma Aldrich Chemical Co., St. Louis, MO, USA).

### 4.10. Data Analysis

Data on flavonoids, phenols and amino acid content were subjected to one-way analysis of variance (ANOVA) and Tukey’s post hoc test at a 95% confidence level to evaluate the effect of the different microwave drying treatments using NCSS 2000 statistical software (NCSS Statistical Software, Kaysville, UT, USA). All analyses were performed in triplicate.

## Figures and Tables

**Table 1:** Microwave (MW) power and treatment time and results for final temperature, weight loss and residual moisture content for the microwave drying tests. Final moisture content are compared with that of the initial untreated pollen sample (UP).

Sample	MW Power (W) ^1^	Treatment Time (min)	Final Temp. (°C)		Weight Loss	Moisture Content ^2^
			Top	Bottom		
UP	–	–	–	–	–	25.50%
MW 150/30	150	30	26.5	36.5	20.9%	6.53%
MW 200/10	200	10	29	42	12.6%	14.89%
MW 200/20	200	20	36	50	20.0%	8.95%
MW 200/30	200	30	43	64	23.9%	3.73%

^1^ Microwave power delivered to the chamber; ^2^ Determined through thermogravimetric analysis.

**Table 2:** Rutin content after the microwave drying tests; results are compared with that of the initial untreated pollen sample UP.

Sample	Rutin ^1^ (µg/g_DM_)	S.E. (µg/g_DM_)	Variation
UP	324.2 a	14.4	–
MW 150/30	336.5 a	14.0	3.8%
MW 200/10	354.2 a	11.6	9.2%
MW 200/20	331.6 a	2.1	2.3%
MW 200/30	352.7 a	18.8	8.8%

^1^ Average value of three independent measures. Within a column, different letters indicate significant differences according to one-way ANOVA followed by Tukey’s test (*p* ≤ 0.05).

**Table 3:** Free proline, total proline, free amino acids and total amino acids content after the microwave drying tests; results are compared with that of the initial untreated pollen sample UP. Pro, proline; AA, amino acids.

Sample	Free Pro ^1^ (mg/g_DM_)	Total Pro ^1^ (mg/g_DM_)	Free/Total Pro	Free AA ^1^ (mg/g_DM_)	Total AA ^1^ (mg/g_DM_)	Free/Total AA (%)	Free Pro/Free AA (%)
UP	19.9 ± 1.7 a	53.3 ± 3.5 a	37.2 b	27.4± 0.6 a	326.3 ± 2.9 a	8.4 b	72.2 a
MW 150/30	18.1 ± 0.1 ab	47.2± 2.5 a	38.4 b	27.3 ± 0.2 a	260.8 ± 9.1 b	10.4 a	66.4 ab
MW 200/10	16.2 ± 0.3 b	39.0 ± 1.1 b	41.4 ab	23.5 ± 0.3 b	247.3 ± 6.8 b	9.5 ab	68.8 ab
MW 200/20	15.0 ± 1.5 b	34.2 ± 0.6 b	43.7 a	23.4 ± 0.3 b	209.4 ± 16.1 c	11.1 a	63.9 b
MW 200/30	17.7 ± 0.6 ab	39.5 ± 0.1 b	44.3 a	26.2 ± 0.6 a	256.7 ± 5.1 b	10.2 a	67.6 ab

^1^ Data represent the average of three independent measures ± SE. Within a column, different letters indicate significant differences according to one-way ANOVA followed by Tukey’s test (*p* ≤ 0.05).

## Abbreviations

The following abbreviations are used in this manuscript:

AA	Amino Acids
DM	Dry Matter
MW	Microwaves
MW XXX/YY	Sample after the MW drying treatment performed at power XXX W for a duration of YY min
SE	Standard error
TGA	Thermo-Gravimetric Analysis
UP	Untreated Pollen
